# White Matter Microstructural Changes as Vulnerability Factors and Acquired Signs of Post-Earthquake Distress

**DOI:** 10.1371/journal.pone.0083967

**Published:** 2014-01-06

**Authors:** Atsushi Sekiguchi, Motoaki Sugiura, Yasuyuki Taki, Yuka Kotozaki, Rui Nouchi, Hikaru Takeuchi, Tsuyoshi Araki, Sugiko Hanawa, Seishu Nakagawa, Carlos Makoto Miyauchi, Atsushi Sakuma, Ryuta Kawashima

**Affiliations:** 1 Division of Medical Neuroimage Analysis, Department of Community Medical Supports, Tohoku Medical Megabank Organization, Tohoku University, Sendai, Japan; 2 Department of Functional Brain Imaging, Institute of Development, Aging and Cancer (IDAC), Tohoku University, Sendai, Japan; 3 International Research Institute of Disaster Science, Tohoku University, Sendai, Japan; 4 Division of Developmental Cognitive Neuroscience, IDAC, Tohoku University, Sendai, Japan; 5 Department of Nuclear Medicine and Radiology, Institute of Development, Aging and Cancer, Tohoku University, Sendai, Japan; 6 Department of Advanced Brain Science, Smart Ageing International Research Center, IDAC, Tohoku University, Sendai, Japan; 7 Japanese Society for the Promotion of Science, Tokyo, Japan; 8 Graduate Schools for Law and Politics, The University of Tokyo, Tokyo, Japan; 9 Department of Psychiatry, Tohoku University Graduate School of Medicine, Sendai, Japan; Vanderbilt University, United States of America

## Abstract

Many survivors of severe disasters need psychological support, even those not suffering post-traumatic stress disorder (PTSD). The critical issue in understanding the psychological response after experiencing severe disasters is to distinguish neurological microstructural underpinnings as vulnerability factors from signs of emotional distress acquired soon after the stressful life event. We collected diffusion-tensor magnetic resonance imaging (DTI) data from a group of healthy adolescents before the Great East Japan Earthquake and re-examined the DTIs and anxiety levels of 30 non-PTSD subjects from this group 3–4 months after the earthquake using voxel-based analyses in a longitudinal DTI study before and after the earthquake. We found that the state anxiety level after the earthquake was negatively associated with fractional anisotropy (FA) in the right anterior cingulum (Cg) before the earthquake (*r* = −0.61, voxel level *p*<0.0025, cluster level *p*<0.05 corrected), and positively associated with increased FA changes from before to after the earthquake in the left anterior Cg (*r* = 0.70, voxel level *p*<0.0025, cluster level p<0.05 corrected) and uncinate fasciculus (Uf) (*r* = 0.65, voxel level *p*<0.0025, cluster level p<0.05 corrected). The results demonstrated that lower FA in the right anterior Cg was a vulnerability factor and increased FA in the left anterior Cg and Uf was an acquired sign of state anxiety after the earthquake. We postulate that subjects with dysfunctions in processing fear and anxiety before the disaster were likely to have higher anxiety levels requiring frequent emotional regulation after the disaster. These findings provide new evidence of psychophysiological responses at the neural network level soon after a stressful life event and might contribute to the development of effective methods to prevent PTSD.

## Introduction

The Japanese earthquake, a severe earthquake with a magnitude of 9.0, hit Japan on March 11, 2011. Many survivors maintain high anxiety levels due to the earthquake aftermath including frequent aftershocks and dispersed radioactive material leaking from nuclear plants[1]. Therefore, even those without posttraumatic stress disorder (PTSD) often require psychological support [2].

The neurological characteristics of subjects with PTSD [3] or stressful life events [4], [5] have been well characterized. Recently, diffusion-tensor magnetic resonance imaging (DTI) [6] was used to investigate white matter structural changes in patients with PTSD [7]–[10], also in healthy survivors of a disaster [11], suggesting the white matter integrity (WMI) changes in the anterior cingulum (Cg). However, a causal relationship with stressful life events remains unclear because of the cross-sectional designs. Detecting neurological underpinnings as a vulnerability factor and the acquired signs of emotional distress soon after stressful life events might contribute to a better understanding of psychological responses to stressful life events and early detection and prevention of PTSD for normal population. A previous study from our lab demonstrated longitudinal changes in grey matter volume from before to after the earthquake [12], suggesting that the reduced volume in the right anterior cingulate cortex before the earthquake was a pre-existing vulnerability factor, and the decreased volume in the left orbitofrontal cortex from before to after the earthquake was an acquired sign of post-earthquake stress. Beyond grey matter volume changes, investigating longitudinal FA changes before and after an earthquake can provide detailed evidence of microstructural abnormalities, particularly in structural connectivity related to emotional distress, after stressful life events.

This study attempted to identify WMI changes representing vulnerability factors and acquired signs of survivors' reports of emotional distress based on a longitudinal study of DTI data obtained from normal subjects before and after the earthquake. In fact, multiple studies performed in our laboratory collected DTI data from a group of healthy subjects before the earthquake. Therefore, this tragedy provided a rare opportunity to investigate WMI changes associated with such a disaster. Thirty subjects were recruited from this group to examine DTI 3∼4 months after the earthquake. Anxiety levels were assessed as a measure of emotional distress following the disaster using the Japanese version of the State–Trait Anxiety Inventory (STAI) [13],[14]. State anxiety represents a psychological response to a stressful event, whereas trait anxiety represents a stable feature of one's personality. Therefore, we assumed that state anxiety scores were more appropriate than trait anxiety scores for assessing the psychological distress experienced soon after the earthquake, and we used state anxiety scores as a measure of the psychological distress experienced soon after the earthquake. We hypothesized that (a) vulnerability factors for anxiety levels after the earthquake could be detected by a significant association between state anxiety and FA before the earthquake (Pre FA) around brain regions previously implicated in PTSD and (b) the acquired signs could be detected by a significant association between state anxiety and WMI changes from before to after the earthquake (Post – Pre FA).

## Methods and Materials

### Recruitment and selection of participants

Eligible right-handed participants with no history of neuropsychiatric disorders were recruited from the undergraduate and postgraduate student population of the Tohoku University community. All candidates had participated in previous Magnetic Resonance Imaging (MRI) experiments conducted in our laboratory, had undergone DTI in the 2 years before the earthquake, and had agreed in advance to re-analyses of MRI scans taken before the earthquake. Because all candidates lived near the city of Sendai, which was seriously affected by the earthquake, control subjects with no experience with the earthquake were not recruited. Screening for neuropsychiatric disorders was conducted using the Mini-International Neuropsychiatric Interview (M.I.N.I.) [15], [16]. Handedness was assessed using the Edinburgh Handedness Inventory [17]. Among the numerous candidates in our database of past experiments, 30 could be contacted. All candidates enrolled in this study were part of a previous study conducted in our lab investigating grey matter volume before and after the earthquake [12]. Among the participants in this study [12], those who did not undergo DTI were excluded from the current investigation. All candidates met the above eligibility criteria and provided written informed consent before participating in the study. The M.I.N.I. confirmed that no subject had a history of psychiatric illness, including PTSD. Additionally, no subjects were taking medications for psychiatric symptoms according to a self-report questionnaire written both before and after the earthquake. This study and all previous studies were approved by the Ethics Committee of Tohoku University School of Medicine.

### Psychological evaluation

All participants were evaluated for levels of anxiety using the STAI [13], [14]. The STAI measures state anxiety levels by asking subjects about their feelings “right now,” whereas it measures trait anxiety levels by asking about their “usual” feelings. Levels of depression were assessed by the Center for Epidemiologic Studies Depression scale (CESD) [18], [19]. Coping styles used in daily life were assessed using the Stress Coping Inventory (SCI) [20]; Japanese version developed by the Japanese Institute of Health [21]. The SCI includes two major factors: 1) cognitive coping strategy, and 2) emotional coping strategy.

All participants were also interviewed by trained psychologists using the Japanese version of the Clinician-Administered PTSD Scale (CAPS) structured interview [22], [23]. In accordance with the M.I.N.I., no subject was diagnosed with PTSD. As for criterion A in CAPS, seven subjects experienced the earthquake as a supra-threshold psychological trauma. Although they did not experience direct life-threatening events due to the earthquake or tsunami, some of them thought that the houses or buildings that they were in at the time of the earthquake might collapse, and some of them thought that the leakage of radioactive materials from nuclear plants might be life threatening. As a result, these seven subjects were assessed to have satisfied criterion A. Actually, four of the seven subjects who met criterion A did not have any PTSD symptoms. As for criteria B, C and D in CAPS, of the 30 participants, seven met more than one criterion but none met all criteria for the three clusters of PTSD symptoms, which include re-experiencing the event, avoidance, and hyperarousal. Four of the seven subjects who had more than one PTSD symptom also did not experience life-threatening events due to the earthquake or tsunami. Specifically, the PTSD symptoms of these four subjects were mainly caused not by the earthquake directly but by the leakage of radioactive materials from nuclear plants or differences in interpersonal relationships after the earthquake. We believe that psychological stress from this kind of disaster comes not only from the disaster itself but also from continuous stressful events after the disaster. Additionally, the highest total CAPS score was 39, which is categorized as subthreshold PTSD [24]. Therefore, all subjects were regarded as “non-PTSD.” The structured diagnostic interview and MRI analysis were conducted 3∼4 months after the earthquake.

All psychological measurements were evaluated after the earthquake. The demographic characteristics of the subjects are presented in Table 1.

**Table 1:** Demographic characteristics of the non-PTSD survivors.

Number of subjects (male/female)	30 (24/6)
Age (years)	21.0±1.6
Number of previous lifetime traumas	1.97±1.0
Period between pre- and post-earthquake MR imaging (days)	271.4±122.9
CAPS	
Total	7.0±11.5
Re-experience	1.7±2.7
Avoidance	2.5±5.1
Hyperarousal	2.8±5.1
CESD score	11.3±10.0
STAI scores	
State	42.9±11.2
Trait	42.5±9.1
SCI scores	
Co	32.7±14.2
Em	30.7±9.8

Values are shown as means ± standard deviations.

CAPS, clinician-administered PTSD scale; CESD, Center for Epidemiologic Studies Depression scale; STAI, State–Trait Anxiety Inventory; SCI, Stress Coping Inventory; Co, cognitive coping strategy; Em, emotional coping strategy.

### Image acquisition

All MRI data were acquired with a 3-T Philips Intera Achieva scanner. The diffusion-weighted data were acquired using a spin-echo echo-planar imaging (EPI) sequence (TR = 10,293 ms, TE = 55 ms, big delta (Δ) = 26.3 ms, little delta (δ) = 12.2 ms, FOV = 22.4 cm, 2×2×2 mm^3^ voxels, 60 slices, SENSE reduction factor = 2, number of acquisitions = 1). The diffusion weighting was isotropically distributed along 32 directions (b value = 1,000 s/mm^2^). Additionally, a dataset with no diffusion weighting (b value = 0 s/mm^2^; b0 image) was acquired. The total scan time was 7 min 17 s. Then, FA values were calculated from the collected images. This information is of particular interest when making inferences regarding white matter microstructural properties, as diffusion is faster along axons than in the perpendicular direction. Consequently, diffusion in white matter is anisotropic (*i*.*e*., diffusion rates in different directions are unequal). By contrast, isotropic diffusion is equally fast in all directions. FA in each voxel was used as a measure of the degree of diffusion anisotropy. FA varies between 0 and 1, with 0 representing isotropic diffusion and 1 representing diffusion occurring entirely in one direction. After DTI image acquisition, FA map were calculated from DTI data using the software that was pre-installed on the Philips MR console.

### Pre-processing of diffusion imaging data and statistical analysis

Pre-processing and data analysis were performed using statistical Parametric Mapping software (SPM5; Wellcome Department of Cognitive Neurology, London, UK) implemented in MATLAB (MathWorks, Natick, MA, USA). First, our original b0 image template was created as follows. Using the affine and nonlinear spatial normalization algorithm, the b0 images from the pre-earthquake scans of all subjects in this study were spatially normalized to the SPM5 T2 template, which is based on averages taken from 152 brains from the Montreal Neurological Institute database. Then, we calculated a mean image of the normalized b0 images as our original b0 image template. Using the affine and nonlinear spatial normalization algorithm, the b0 image of each participant was normalized to our original b0 image template. Before normalization of the FA map, the post-earthquake FA maps were co-registered with the pre-earthquake FA maps from each subject. Then, using the parameter for this affine and nonlinear normalization procedure, an FA map of each participant was spatially normalized to yield images with 2 × 2 × 2-mm voxels and spatially smoothed using a Gaussian kernel of 10 mm FWHM. The resulting maps representing the FA were then subjected to the group regression analysis described below.

### Statistical analyses

The group-level analysis tested for a relationship between individual state anxiety as measured by the STAI and regional FA. Voxel-by-voxel multiple regression analyses were performed using the state anxiety for Pre FA and Post–Pre FA in VBM5 on SPM5. The analysis was performed with sex and period between the pre- and post-earthquake MRI data acquisition as additional covariates. According to the relevant guidelines[25], this study, which had a sample size of 30, should be limited to two variables. Therefore, we made it a priority to control the effects of sex and period, and age was not included in the analysis as a covariate. All tests of FA were performed using an absolute threshold of FA >0.2 [26], such that if a voxel anywhere in the brain had an FA value >0.2 in all subjects, that voxel was included in the analysis. This measure was used because FA is more susceptible to errors arising from partial volumes [27], and this FA cut-off value allowed us to dissociate white matter structure from other tissue [28].

Significant regions were inferred using cluster-level statistics [29]. In this procedure, the null hypothesis was rejected when the clusters had a large spatial extent. The distribution of cluster sizes was found by parametric methods based on the theory of Gaussian random fields, which accounts for image volume, smoothness, and the cluster-defining threshold. At the cluster level, inference is determined according to the cluster size; that is, the probability that any cluster is larger than the critical cluster size is controlled. Only clusters with a *p*-value <0.05 after correction for multiple comparisons related to cluster size and an uncorrected voxel-level cluster-determining threshold of *p*<0.0025 were considered statistically significant in this analysis [30]. Next, to evaluate the strength of the association between white matter structural changes and state anxiety levels, we performed structural equation modeling (SEM) using the state anxiety scores from the STAI, Pre FA, and Post–Pre FA at peak voxels in each cluster as observed variables. Finally, we performed *post hoc* correlation analyses between the FA values in the regions of interest (ROIs) found in the aforementioned whole-brain analyses for trait anxiety scores, CAPS, and the two main factors of the SCI.

## Results

The demographic characteristics of subjects are presented in Table 1. The distribution of anxiety levels is illustrated in Table 2. The state anxiety scores show significant positive correlations with both trait anxiety (r = 0.66, *p* = 0.0001) and CAPS scores (r = 0.50, *p* = 0.005). We also found a significant negative correlation between the state anxiety scores and the factor representing emotional coping strategy on the SCI (r = −0.54, *p* = 0.002).

**Table 2:** Distribution of anxiety levels.

	Extremely low	Low	Normal	High	Extremely high
State	0	5	10	10	5
Trait	0	3	16	6	5

STAI, State–Trait Anxiety Inventory.

**Figure 1:**
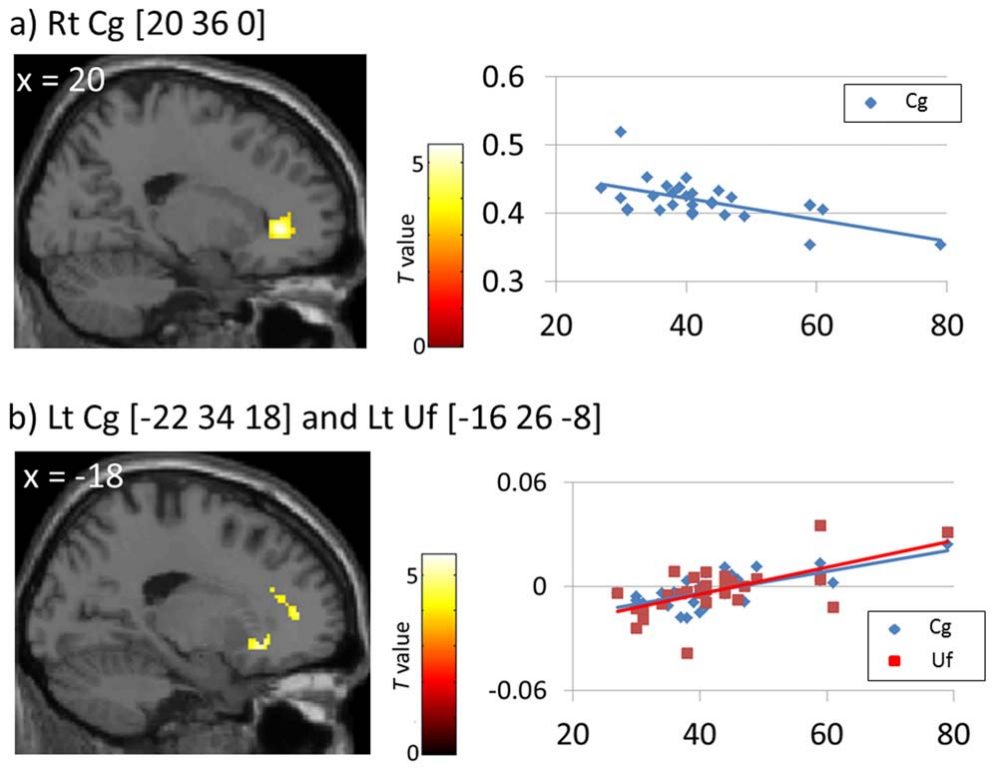
Relationship between state anxiety and FA. State anxiety scores were negatively associated with Pre FA in the right anterior Cg (a, *r* = −0.61, *p* = 0.0004) and Post–Pre FA in the left anterior Cg (*b*, *r* = 0.70, *p* = 0.00002) and the left Uf (*b*, *r* = 0.65, *p* = 0.0001), as illustrated by the scatter plots on the right. Vertical axes represent FA values at peak voxels in each cluster and horizontal axes indicate total state anxiety scores. The left Uf and the Ac were included in the same cluster. FA, fractional anisotropy; Rt, right; Lt, left; Cg, cingulum; Uf, uncinate fasciculus.

**Figure 2:**
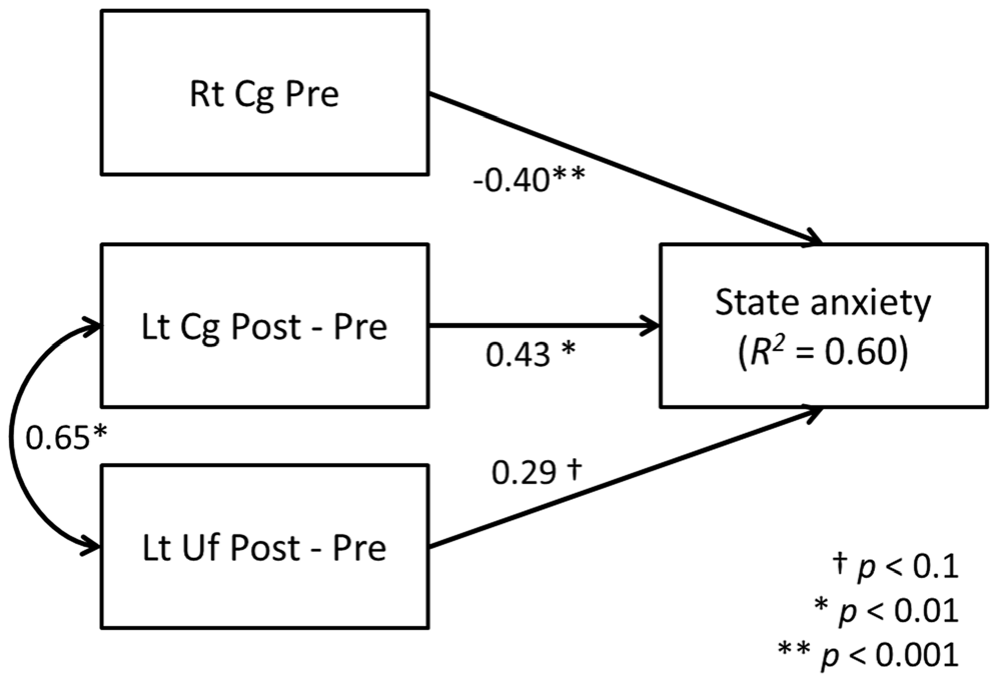
SEM implemented on a path diagram. The strength of the path coefficients between state anxiety scores and Pre FA in the right anterior Cg (−0.40, *p*<0.001) and between state anxiety scores and Post–Pre FA in the left anterior Cg (0.43, *p*<0.01) and the left Uf (0.29, *p* = 0.06) are shown. The path coefficient strength between Post–Pre FA in the left anterior Cg and the left Uf (0.65, *p*<0.005) are shown as well. The brain regions that predict state anxiety level evaluated by STAI are shown on the left. FA, fractional anisotropy; Rt, right; Lt, left; Cg, cingulum; Uf, uncinate fasciculus; STAI, State–Trait Anxiety Inventory.

After controlling for sex and the period between pre- and post-earthquake MRI data acquisition, state anxiety scores were negatively associated with Pre FA in the right Cg (Montreal Neurological Institute [MNI] coordinates, x = 20, y = 36, z = 0; Fig. 1a, Table 3) and positively associated with Post–Pre FA in the left anterior Cg (MNI coordinates, x = −22, y = 34, z = 18; Fig. 1b; Table 3) and with a cluster including both of the left uncinate fasciculus (Uf; MNI coordinates, x = −18, y = 26, z = −8; Fig. 1b, Table 3) and the anterior commissure (Ac; MNI coordinates, x = −10, y = 18, z = −8; Table 3). Furthermore, SEM data showed that Pre FA in the right anterior Cg and Post–Pre FA in the left anterior Cg and the left Uf accounted for 60% of the score variance in state anxiety (*R*2 = 0.60; Fig. 2). Additionally, the *post hoc* correlation analysis revealed that the Post–Pre FA in the left Uf was negatively correlated with the factor of cognitive coping strategy (*r* = −0.40, *p* = 0.029) and emotional coping strategy on the SCI (*r* = −0.44, *p* = 0.015). Also, the trait anxiety scores were negatively correlated with the Pre FA in the right Cg (*r* = −0.47, *p* = 0.010) and positively correlated with the Post – Pre FA in the left Cg (*r* = 0.42, *p* = 0.022) and the left Uf (*r* = 0.36, *p* = 0.049). No significant correlations were observed between the FA values in each ROI and CAPS scores.

**Table 3:** MNI coordinates, voxel sizes, *z*-scores, and *P*-values for results of the SPM analyses.

Brain region	MNI coordinates	k (voxels)	*z*-scores	*p*-values
	x y z			(cluster level)
Pre				
Rt Cg	20 36 0	310	4.62	0.010
Post–Pre				
Lt Cg	−22 34 18	128	4.34	0.026
Lt Uf	−18 26 −8	161	4.12	0.013
Ac*	−10 18 −8		3.83	

MNI, Montreal Neurological Institute; Rt, right; Lt, left.

Cg, cingulum; Uf, uncinate fasciculus; Ac, anterior commissure.

*The Ac is included in the same cluster as the Lt Uf.

## Discussion

State anxiety scores were negatively associated with Pre FA in the right anterior Cg and positively associated with Post–Pre FA in the left anterior Cg, the left Uf, and the left Ac. According to our hypothesis, lower WMI in the right anterior Cg and increased WMI in the left anterior Cg, the left Uf, and the left Ac were white matter structural changes that respectively represented vulnerability factors and acquired signs of anxiety level after the earthquake.

Several lines of evidence support the notion that lower WMI in the right anterior Cg is a vulnerability factor for anxiety after stressful events. The anterior Cg bundle is a part of the principal white matter tract in the Papez circuit, which includes the ACC and the amygdala [31]. Decreased anterior Cg WMI in patients with PTSD has been frequently reported [8]–[10]. Also, smaller dorsal and ventral ACC volumes are reported in patients with PTSD [32], [33] and in normal subjects after stressful life events [4]. Regarding vulnerability factors, a previous study from our lab found that a smaller right ventral ACC volume was a pre-trauma vulnerability factor for PTSD symptoms [12], which is congruent with the present findings. On the other hand, investigations of monozygotic twin pairs with PTSD have found that smaller hippocampal volume was a vulnerability factor [34] and that a smaller rostral ACC was an acquired sign of PTSD [35]. Although there are apparent discrepancies between our findings and the monozygotic twin studies, it is postulated that the discrepant findings result from fundamental differences in study designs. Monozygotic twin studies cannot distinguish acquired signs of PTSD from acquired signs from birth to trauma because of the cross-sectional design of the study, which occurs after the traumatic events [35]. Based on our findings, the lower WMI in the right anterior Cg is a pre-trauma vulnerability factor for anxiety levels after a stressful event. This may have been identified as an acquired sign in the monozygotic twin study.

The functional roles of the anterior Cg and Uf indicate that psychological responses of survivors occur soon after the earthquake. The Uf, also involved in the emotional processing [36], is a principal white matter tract that connects the orbitofrontal cortex (OFC) and limbic regions including the amygdala and the anterior temporal cortices [37] [38]. In fact, neural responses in the OFC are preferentially enhanced with those in the amygdala during extinction [39] and this relationship is crucial to the voluntary regulation of emotion [40]. Given our finding that scores for cognitive coping strategy were negatively correlated with scores for increased FA in Uf, those who were unlikely to have a cognitive coping strategy in daily life may have increased their WMI in Uf soon after the earthquake; this may have been induced by frequent reliance on emotional regulation due to post-earthquake stress. Based on previous cognitive training studies suggesting that the integrity of the white matter related to trained cognitive functions increases [41], [42], this would be expected to strengthen the integrity of the white matter. Therefore, subjects with the increased WMI in the left Uf and Cg would be required to regulate their emotions more frequently, but failed to regulate. Then they had higher state anxiety levels than subjects with decreased WMI in the left Uf and Cg.

In contrast, there is an apparent discrepancy with previous DTI studies demonstrating lower WMI in the anterior Cg and/or the Uf in patients with anxiety disorders such as PTSD [8]–[10], social anxiety disorder (SAD) [43], and generalized anxiety disorder (GAD) [44] and in healthy subjects with high anxiety levels [45], [46] [11]. It was suggested that lower WMI in the Cg and/or Uf represents a dysfunction of emotion regulation in patients with anxiety disorders [8]–[10], [43], [44]. We believe that this discrepancy between increased and lower WMI could be explained by a difference in early stage and long lasting anxiety levels. High anxiety levels soon after a stressful life event would be associated with frequent access to the anterior Cg and the Uf cognitive functions, which are involved in emotional processing and emotional regulation, respectively. Conversely, long lasting high anxiety levels, which is also common in the aforementioned anxiety disorders, induces cognitive dysfunction, which is associated with lower WMI in the anterior Cg and Uf. This interpretation is consistent with that of diffusional anisotropy elevation caused by temporary activation of the Cg in PTSD [7], which is also supported by our findings that state anxiety scores, which represent possibly temporary anxiety levels experienced soon after the earthquake, were more strongly correlated with increased WMI in these regions than were trait anxiety and CAPS scores. Together, these findings indicate that increased WMI in the anterior Cg and the Uf represents early-stage psychological responses to a stressful life event, and decreased WMI represents the late stage, which is reflected in the development of anxiety disorders (*e*.*g*., PTSD, SAD, and GAD).

In addition, these findings indicate asymmetrical characteristics of anterior Cg psychological responses to a stressful life event in normal subjects. Previous neuroimaging studies investigating patients with PTSD revealed right hemisphere predominance [47], left hemisphere dysfunction [48], and asymmetrical WMI reduction in the anterior Cg [9]. Asymmetrical functional connectivity in the cognitive division of the ACC exists in healthy subjects as well [49]. A possible interpretation of the current results is that those with low right anterior Cg function are likely to become anxious but are protected against the development of PTSD by the maintenance of left anterior Cg function.

Some limitations should be considered when interpreting our results. First, psychological data related to emotional distress, such as anxiety levels before the earthquake, were not available. This was a predetermined limitation of the study because pre-earthquake dataset were not obtained to address the emotional issues. Additionally, because the STAI is designed to assess general anxiety in a non-specific manner, we could not determine whether the anxiety levels were caused by the earthquake. However, all subjects had no history of psychiatric diseases, suggesting their anxiety levels before the earthquake were within normal levels. Also, the significant correlation between state anxiety levels and CAPS scores indicates that the anxiety levels were raised by the earthquake, because CAPS scores were definitely results of the earthquake. Furthermore, the SEM analysis supports the model in which the direction from brain structural changes to anxiety levels was suggested. The results of these analyses complement the lack of psychological data before the earthquake. Second, this study did not include subjects with supra-threshold PTSD symptoms, because most candidates in our pre-earthquake database were assumed to have been affected by the earthquake to some extent but not to have been exposed to life-threatening experiences. Therefore, the scope of the current study was the neural correlates of individual differences in state anxiety levels in the normal population after experiencing the disaster, regardless of psychological trauma. We believe that investigation of subjects with subclinical PTSD symptoms can provide sufficient evidence, an assumption that has been made in previous studies [4], [5], [11], [50], [51], [52], which would namely contribute to early detection and prevention of PTSD. In any case, a further longitudinal study of patients with supra-threshold PTSD symptoms caused by traumatic events is necessary to examine whether the neural microstructural connectivity changes observed in the current investigation are applicable to such individuals. Third, the majority of our subjects were males. To deal with this issue, sex was treated as an additional covariate. However, the possibility that the unbalanced sex distribution of subjects distorted the results remains. Fourth, the participants in the present study were limited to university students. Thus, the results may not generalize to older populations.

Despite these limitations, this is the first longitudinal study distinguished WMI changes that represent a vulnerability factor from structural changes that represent an acquired sign of high state anxiety. Additionally, the results demonstrating increased FA in the left anterior Cg and the Uf provide new evidence of temporal FA elevation in the early-stage response to stressful life events before anxiety disorders (*e*.*g*., PTSD, SAD, and GAD) develop. Such disorders are characterized by decreased FA in these areas. These findings may be helpful for discriminating between survivors with and without emotional distress soon after a stressful life event, and between survivors who will and will not, in future, experience anxiety after a stressful life event, even in the normal population. These findings provide a better understanding of psychophysiological responses to a stressful life event at the neural network level and may contribute to the development of effective methods to prevent stress-related disorders, namely PTSD, in the normal population.
